# A Novel Segmentation, Mutual Information Network Framework for EEG Analysis of Motor Tasks

**DOI:** 10.1186/1475-925X-8-9

**Published:** 2009-05-04

**Authors:** Z Jane Wang, Pamela Wen-Hsin Lee, Martin J McKeown

**Affiliations:** 1 Department of Electrical and Computer Engineering, University of British Columbia, Vancouver, BC, Canada; 2 Pacific Parkinson's Research Center, University of British Columbia, Vancouver, BC, Canada

## Abstract

**Background:**

Monitoring the functional connectivity between brain regions is becoming increasingly important in elucidating brain functionality in normal and disease states. Current methods of detecting networks in the recorded electroencephalogram (EEG) such as correlation and coherence are limited by the fact that they assume stationarity of the relationship between channels, and rely on linear dependencies. In contrast to diseases of the brain cortex (e.g. Alzheimer's disease), with motor disorders such as Parkinson's disease (PD) the EEG abnormalities are most apparent during performance of dynamic motor tasks, but this makes the stationarity assumption untenable.

**Methods:**

We therefore propose a novel EEG segmentation method based on the temporal dynamics of the cross-spectrogram of the computed Independent Components (ICs). We then utilize mutual information (MI) as the metric for determining also nonlinear statistical dependencies between EEG channels. Graphical theoretical analysis is then applied to the derived MI networks. The method was applied to EEG data recorded from six normal subjects and seven PD subjects off medication. One-way analysis of variance (ANOVA) tests demonstrated statistically significant difference in the connectivity patterns between groups.

**Results:**

The results suggested that PD subjects are unable to independently recruit different areas of the brain while performing simultaneous tasks compared to individual tasks, but instead they attempt to recruit disparate clusters of synchronous activity to maintain behavioral performance.

**Conclusion:**

The proposed segmentation/MI network method appears to be a promising approach for analyzing the EEG recorded during dynamic behaviors.

## Background

Connectivity between brain regions is important for normal brain functioning, and may be impaired in many neurological diseases [1]. The electroencephalogram (EEG), with its excellent temporal resolution (~1 msec), is the most widely available technology used for inferring transient synchronization between brain regions. Both linear and nonlinear measures have been applied to assess the interdependencies between EEG channels [2]. For example, coherence and correlation methods [3,4], which measure the dependencies between a pair of EEG signals in the frequency and time domains respectively, have been applied to the EEG to study the cortical synchrony that can be modulated as a function of task, and may systematically differ between normal and disease groups [5,6]. Nevertheless, these measures consider only linear dependencies and may be particularly sensitive to outliers. Other methods may also be used to investigate both linear and non-linear relationships between multivariate time series in the EEG, such as the Synchronization Likelihood (SL), but this and related methods assume a fixed phase relationship between time series [7]. However, in some diseases such as PD, transient phase-locked behavior between different parts of the motor system may be interrupted by "phase slips" [8] making the assumption of prolonged periods of phase synchrony potentially unsuitable.

An alternative to the linear methods of coherence and correlation and phase synchronization is to consider the mutual information (MI) between channels within a specified window. This enables estimation of both the linear and nonlinear statistical dependencies between time series and can be used to detect functional coupling. MI is a statistical technique that quantifies the information transmitted from one time series to another, with maximum value when two time series are the same and a value of zero if two time series are statistically independent. Previously, researchers have utilized MI as a suitable metric to investigate EEG coupling in various pathological conditions [9-11]. For example, by estimating the MI between the time series of multiple pairs of EEG channels, Jeong et al. demonstrated abnormalities in the information transmission between different cortical areas in Alzheimer's patients [9]. Similar studies have used MI as a marker for cortico-cortical connections in schizophrenic patients [10] and odor stimulation [11].

Another disease where altered connectivity may be important is Parkinson's disease (PD), a movement disorder that is characterized by muscle rigidity, tremor, and bradykinesia (slowing of physical movement). These symptoms do not reflect a primary failure of the cortex (making resting EEG less likely to be abnormal), but rather the effects of failure of the basal ganglia to prime the cortex for preparation and execution of movement. As a result, PD patients have a difficult time performing simultaneous movements compared to normal subjects [12,13]. In order to assess the indirect effects of basal ganglia dysfunction on the cortex in PD, it is necessary to have subjects perform a motor task. Furthermore, stressing the motor system by having PD subjects performing simultaneous movements is more likely to induce abnormalities in the recorded EEG.

However, as soon as a subject performs a dynamic motor task, the non-stationarity nature of the EEG must be considered [14]. The non-stationarity likely reflects the switching of inherently metastable states of neural assemblies during task performance causing abrupt transitions. The non-stationary property of EEG suggests that techniques assuming stationarity may result in misleading interpretations. To address this concern, a conventional approach is to incorporate a sliding time window into the original signal models, and assume that the stationarity assumption is valid for the segment of data in the window. However, the selection of an appropriate (possibly time-varying) window length is non-trivial and could have a significant effect on the analysis results.

In order to obtain quasi-stationary segments in EEG signals and select task-related segments, we first propose a novel segmentation method of the EEG based on the temporal dynamics of the cross-spectrogram of the Independent Components (ICs), and then compute the MI between channels within the temporally-segmented regions. We then apply graph theoretical analysis to the network of each group defined by edges whose MI values exceed a suitable threshold, and compute the clustering coefficient (*C*) and shortest path length (*L*) [15,16]. In order to accommodate the magnitude of MI values above a given threshold, the intra-group (ie. task) and inter-group (ie. subject groups) network differences are further analyzed by one-way analysis of variance (ANOVA).

For motor tasks, changes in the EEG are most likely related to event-related synchronization/desynchronization (ERS/ERD), particularly in the beta band [17]. In self-paced movements, ERD corresponds to changes in coherence between brain regions [18]. Thus we suggest the use of transient synchronization of between ICs as suitable markers for segmentation [19,20]. We emphasize that the concept of "task-related sections" is flexible and may be dependent upon the underlying behavioral paradigm subjects are asked to perform. For example, if a subject was asked to push a button every 10 seconds, then transient synchronization of ICs occurring approximately every 10 seconds may be a suitable marker for segmentation. Here we demonstrate the proposed segmentation method in a less obvious situation: ongoing modulation of continual manual force production.

To our knowledge, this is the first application of using transient synchronization of ICs for temporal segmentation of time series. Also, it is the first application of joint MI and network analysis to assess information transmission abnormalities between different cortical areas in PD. The main contributions of this paper are as follows:

• Propose a novel EEG segmentation approach to address the non-stationary nature of EEG data especially during performance of motor tasks, and to select task-related segments.

• Present a coherent MI-based network analysis framework for modeling EEG to determine dependencies between EEG channels and infer statistically significant difference between groups.

• Demonstrate how the proposed framework can be used for assessing the EEG during dynamic motor behaviors in pathological conditions, such as PD.

The paper is organized as follows: the detailed discussion of the proposed framework is presented in the Methods section. The Results and Discussion section describes the EEG experimental design and summarizes the results in a real case study of PD. Finally, we summarize and conclude the paper in Conclusions.

## Methods

We recorded five minutes of EEG data while subjects performed simple hand movements in order to gain insight into the difficulty that PD subjects face when performing simultaneous movements.

### Preprocessing

**Figure 1:**
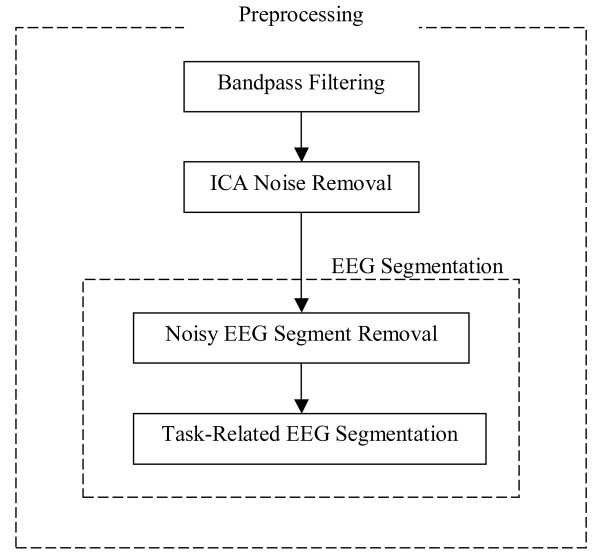
**Flowchart diagram of the steps of EEG preprocessing**.

Fig. 1 presents the flowchart diagram of the steps of EEG preprocessing. The preprocessing steps include bandpass filtering to focus on the frequency range of clinical interest, Independent Component Analysis (ICA) for artifact removal in EEG, and EEG segmentation based on the cross-spectrogram of the ICs to address the non-stationary nature of EEG data and to select task-related segments. At the beginning of each step, we briefly describe the motivation for selecting the respective method.

#### Bandpass Filtering

EEG data contain a wide range of frequency components, many of which are not of clinical or physiological interest. The data are therefore initially bandpass-filtered by a 4th order Butterworth filter between 0.5–55 Hz [21].

#### ICA Noise Removal

ICA has been proven capable of isolating both artifactual and neurally generated EEG sources [22]. As various contaminants of EEG recordings such as eye movements, eye blinks, cardiac signals, muscle contamination, etc., can be considered temporally independent from ongoing brain activity, ICA is a popular class of methods for EEG de-noising and artifact removal in EEG. ICA decomposes mixtures of time courses into a sum of temporally statistical maximally independent components. The EEG measurements from the scalp, *x *= {*x*_1_(*t*),..., *x*_*N *_(*t*)}, are mixtures of the source signals, *s *= {*s*_1_(*t*),..., *s*_*N *_(*t*)}, where *N *is the number of EEG channels and *t *denotes the time. The task of ICA is to recover a version, *u*, of the original sources, *s*, by finding an unmixing matrix, *W*, specifying spatial filters that invert the mixing process linearly, as

(1)

Here the infomax-ICA algorithm [23] is applied to decompose the EEG signals. ICA finds a coordinate frame in which the data projections have minimal temporal overlap by minimizing the mutual information among the data projections or maximizing the joint entropy of a nonlinear function of *s*. It is most appropriate to perform ICA decomposition on sources that are linearly mixed in the recorded signals without time delays. After the artifactual sources are identified, the corresponding columns of the mixture matrix (i.e. calculated as the pseudo-inverse of *W*) that multiply the artifactual sources are set to zero to eliminate the artifacts and thus obtain the "corrected" EEG signal. In our study, we only remove well-known, obvious artifacts by identifying 1 to 2 components (e.g. representing eye-movements and/or electrocardiac signals) by visual inspection. Failure to remove these artifacts may result in correspondence between EEG channels being falsely attributed to synchronized brain activity.

#### EEG Segmentation based on the Cross-Spectrogram

**Figure 2:**
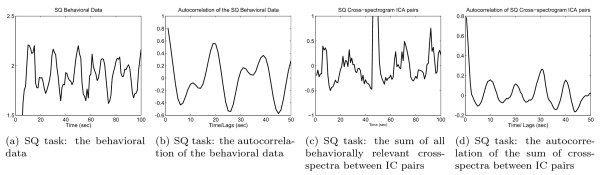
**An example of the original and autocorrelation of the Squeeze (SQ) behavioral data and the sum of cross-spectrogram Independent Component (IC) pairs integrated between 8–12 Hz**. Both the autocorrelation of the behavioral data and the autocorrelation of the integrated cross-spectrogram IC pairs contain peaks around every 10 and 18–20 seconds, yet there are some discrepancies between their actual time courses.

Since the EEG contains much background brain activity that may be unrelated to the motor task being performed, it is necessary to segment the data into task-relevant sections. The task-relevant sections are those segments of the EEG data that correspond to the underlying experimental motor task being performed by the subject. The motor task here is designed to target the relative difficulties PD subjects have with performance of simultaneous movement compared. The experimental design is explained in detail in the section Results and Discussion-A. Another purpose of segmentation is to address the non-stationary nature of EEG data [14] by achieving local stationarity. Since final motor output is dependent upon cortical, subcortical, brainstem and spinal circuits yet the EEG measures only cortical activity, segmentation of the EEG based on behavioral data alone may be misleading (Fig. 2). We therefore propose segmenting the EEG based on the cross spectrum of task-related ICs.

We note that if the derived ICs were truly independent, then the cross-spectrum would not be significant. However, in real data many of the assumptions of ICA are violated. The data are not stationary, and the time courses are not temporally white. By using infomax-ICA, which does not incorporate time delays, the derived components will be maximally independent at zero lag. As such, it will deal with the problem of volume conduction – where a deep electrical source may impart common electrical activity to two or more channels. Even though ICs are maximally independent over the whole time range, they may exhibit partial synchronization within specific time/frequency window [24], through which the transient coupling of neural networks might be revealed. By examining the ICs within a short moving window, the non-stationary nature of the EEG will be explored, and significant dependencies between ICs become apparent. Recent studies such as [19,20] have also explored the transient synchrony between ICs and suggested transient correlation between ICs.

The ICs are thus transformed into time-frequency domain and the cross-spectrogram of every pair of ICs is computed. The frequency contents are computed by cross power spectral density using the Welch's averaged, modified periodogram [25] method of spectral estimation. Suppose {*x*(*k*)} and {*y*(*k*)} are real sequences with length *N *normalized to zero mean and unit variance, their cross-correlation sequence is defined as:

(2)

The cross spectral density function *S*_*xy*_(*f*) is the Fourier transform of the cross-correlation sequence {*R*_*xy *_(*m*)}, expressed as,

(3)

with *f *being the frequency normalized by the sampling frequency. By using a short-term time shifting window, we are able to obtain localized frequency contents of the two signals and their relationship with respect to time and frequency. The cross-spectrum is computed based on a short (3 s) time window shifted by 0.5 s to obtain the localized time information. Power in the higher frequency ranges, such as the gamma band (> 20 Hz) are more likely to distributed over a broad frequency range. To avoid any potential confounds from the AC current at 60 Hz, we look for sharp increases in activity in the range 45 Hz–55 Hz as a good marker for transient broadband artifacts that were not eliminated by the ICA Noise Removal step. In contrast, by examining the cross-spectrogram of pairs of ICs within the lower frequency bands of physiologic interest, we can identify task-related segments.

**Figure 3:**
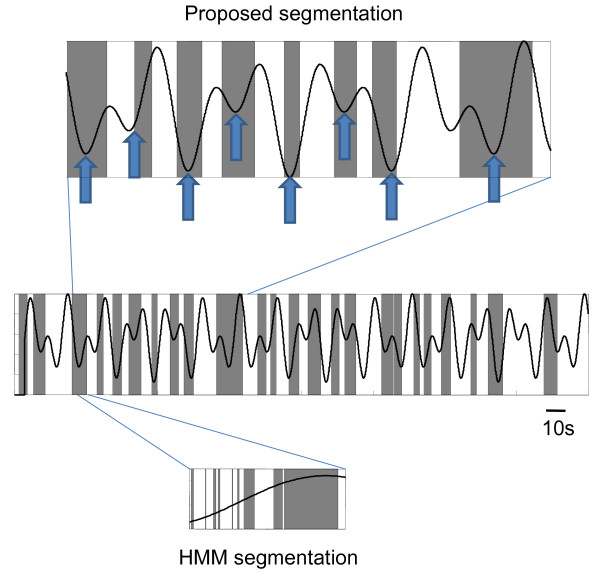
**Proposed segmentation compared to behavioral data**. The sections of EEG segmented out by the proposed technique (shaded boxes) is shown with overlaid force pressure required by the subject during the motor task(s). Segmented sections tend to be around times where there is a reversal of force and an increase in force required (block arrows, top panel), but this is not entirely consistent for the entire data set (middle panel), suggesting that segmenting solely based on the behavioral paradigm might be misleading. In contrast, a HMM-mAR technique results in rapidly cycling between states with no discernable relationship to the behavioral data (bottom panel). For this subject the proposed technique isolated 24 distinct segments, but the HMM-mAR method isolated 418 distinct segments.

We compared the proposed approach of examining the cross-spectra between ICs to a commonly used approach, Hidden Markov Models (HMM), a probabilistically tractable and robust way of modeling the dynamic changes of state. We coupled the HMM framework with multivariate Autoregressive (mAR) models, an approach that has been previously suggested for examining non-stationary multivariate electrophysiological signals [26,27]. Our results suggest that the proposed scheme provides a more reasonable segmentation performance (Fig. 3). In contrast, the HMM-mAR technique resulted in rapidly cycling between states with no discernable relationship to the behavioral data (Fig. 3). Thus, after appropriate segmentation, the EEG sections were then concatenated and a mutual information network was derived.

### Mutual Information based Network

MI measures the mutual dependence or information gained about one signal from another. The detailed derivation and background of information theory can be found in [28]. Given two random variables *X *and *Y*, the pair-wise MI is defined as

(4)

where *P*_*X*_(*x*) is the probability that *x *is drawn from *X *and *P*_*XY *_(*x*, *y*) is the joint probability density function for the measurements of *X *and *Y *that produce the values *x *and *y*.

MI quantifies the amount of information about *X *that *Y *contains. It is a symmetric function meaning *I*(*X*, *Y*) = *I*(*Y*, *X*). A MI at zero means that Y does not contain additional information about X, because *P*_*XY *_(*x*, *y*) factorizes to *P*_*X*_(*x*)*P*_*Y *_(*y*) resulting in MI being zero. On the contrary, the higher the MI between two signals, the more information they contain about each other. Hence, the higher MI, the more likely that the two signals are biologically related. MI is estimated from a finite number of samples, the probability densities, *P*_*X*_(*x*) and *P*_*XY *_(*x*, *y*), are approximated by histogram (using bin size of 20). For a fair comparison across subjects, in our paper we have used the relative MI as

(5)

where *I*_*r*_(*X*, *Y*) is in the range [0, 1], and *H*(*X*) and *H*(*Y*) are the entropies. Entropy *H*(*X*), defined as-Σ_*x *_*P*_*X*_(*x*)*log*_2_*P*_*X*_(*x*), is regarded as a measure of uncertainty about a random variable *X*.

**Figure 4:**
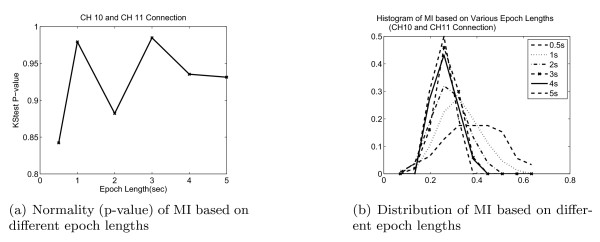
**Relationship between epochs length and normality**. The data is more Gaussian for epoch length between 1 to 4 seconds observed both from the p-value of the KS-test and the distribution of the data.

In our study, the data are separated into 4 second epochs for MI computation in order to increase the sample size as well as to enhance the stationarity and consistency of the MI estimates. Preliminary work of varying the length of the epochs suggested that 4-s epochs give a more Gaussian distribution (Fig. 4(a), (b)).

### Network Analysis

In order to graphically represent a large set of data, we have derived both a relevance network [29] and an MI network. A relevance network, originally devised for graphically depicting the relationship between genes [29], can be generalized to take large data sets of experimental data and graphically depict the result of pair-wise MI. It is obtained by applying a threshold and only the connections that are above the threshold are displayed in the network. The relevance network can then be used in graphical theoretical analysis (discussed in Methods – Network Analysis).

In addition, we have also taken into account the magnitude of MI values and obtained an MI network from the one-way ANOVA test (with details given in Methods – Network Analysis (Statistical Analysis)). The connections are established in the MI network if their MI values exceed a specified threshold and the ANOVA tests indicate significantly different values between groups.

#### Graphical Theoretical Analysis on Relevance Network

Graph theoretical analysis is applied to the MI matrices of all possible pair-wise combinations of EEG channels. The resulting MI matrices are converted to binary relevance networks/graphs by applying a threshold. Graphs are characterized by a cluster coefficient, *C*, and a characteristic shortest path length, *L*. A graph *G *= (*V*, *E*), consisting of a set of vertices *V *(channels) and a set of edges *E *(connections) between the vertices, is a basic representation of a network. An edge *e*_*ij *_connects vertex *i *with vertex *j*. The neighborhood *N*_*i *_for a vertex *v*_*i *_is defined as its immediately connected vertex neighbors. The graph degree *k*_*i *_of vertex *i *is the number of edges linking vertex *i *to its neighbors. The cluster coefficient *C*_*i *_for a vertex is thus defined as the ratio of the number of edges between the neighbors of vertex *i *and the maximum possible number of edges between *k*_*i *_neighbors of vertex *i*. It is defined as

(6)

where |.| means the number of edges included in {*e*_*jk*_}. The cluster coefficient *C *of a graph (the whole system) is defined as the mean cluster coefficient:

(7)

with *n *being the total number of vertices in the graph. Such *C *measures the local connectivity and ranges from 0 to 1. The higher the *C*, the greater the intensity of connections within a cluster.

The *L *of a graph is the mean of all shortest paths (shortest distance) connecting all pairs of vertices. It has a value greater than 1 and measures the global connectivity of the graph. A detail graphical explanation of a graph and graph theoretical measures can be found in [16].

**Figure 5:**
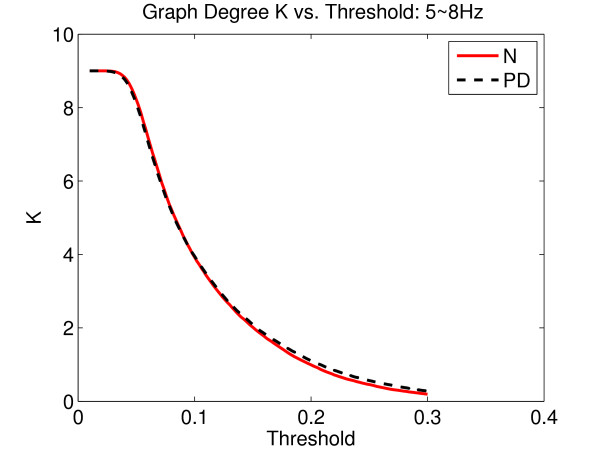
**An illustration of the graph degree *K *as a function of threshold *T***. It is noted that *K *as a function of *T *is approximately the same for all cases.

We computed the *C *and *L *of a graph as a function of a threshold, *T*, ranging from 0.01 to 0.3 with an increment of 0.002 to determine the graph differences between the two groups. Since the results might be biased by the mean level of MI, the graph degree, *K*, defined as the average number of edges per vertex, may be a more suitable measure. However, since the relationship between *K *and *T *were almost identical between groups (Fig. 5) any differences in *C *and/or *L *at the same level of *T *would reflect the actual differences in graph organization.

#### Statistical Analysis

In addition to the graphical theoretic analysis on the thresholded matrices, we created an "MI network" which also incorporated the magnitude of the MI values. As before, MI values were first thresholded by zeroing values less than the 95th percentile on a null distribution. A null distribution was obtained by repeatedly (n = 100) randomly permuting the order of the second signal and computing the pair-wise MI based on them [30]. The MI differences between segments are analyzed by one-way ANOVA with subject number, groups, and tasks as factors [31]. The connections between any two channels are established for the MI network if they are significantly different according to the ANOVA test and have magnitudes that are greater than the permutation threshold. The normality of the distribution of the MI values is verified by the Kolmogorov-Smirnov (KS) test [31].

In the one-way ANOVA test for each pair-wise MI *I*(*X*, *Y*), the effect of a factor (e.g. Group) is tested, by comparing with the F-test the variance of *I*(*X*, *Y*) explained by the factor against the variance of the residuals. Consequently, a *p*-value was calculated for each possible connection in the MI network. To account for the effect of testing multiple connections simultaneously, the *p*-values are corrected for multiple hypothesis testing using Storey's positive-false-discovery-rate (pFDR) procedure [32] which computes a *q*-value, the expected ratio of falsely rejected hypotheses among all those being rejected. Connections whose *q*-values were smaller than 5% are considered statistically significant.

Because we are more interested in the connection with greater MI values, the permutation test is used in conjunction with the ANOVA test to select the relevant features for the MI based network. We have chosen the largest observed value of the permutation test as our threshold. A connection is thus established in the MI network if the MI values are significantly different based on the *q*-value and are above the maximum observation from the permutation test.

## Results and Discussion

### Subjects and Experiment Design

All research was approved by the University of British Columbia Ethics Board. After giving informed consent, seven PD and six age-matched control subjects volunteered to participate in the study. All patients were diagnosed with mild to moderately severe PD (Hoehn and Yahr stage 1–3) [33]. The control subjects were confirmed to be without active neurological disorders by a qualified neurologist. All patients were taken off L-Dopa medication after overnight withdrawal of at least 12 hours.

**Figure 6:**
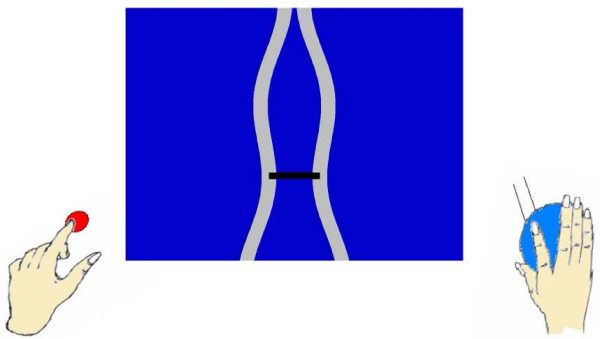
**Experiment design of Squeeze (SQ) and Both (BO) task**. In the middle is the "inflatable" ring indicated by the horizontal bar. Two separate tasks are performed: 1) control the "inflatable" ring by squeezing the bulb with right hand alone; 2) press a mouse button intermittently with their left hand in addition to doing the squeezing with the right hand.

Subjects were asked to hold a custom-built rubber squeeze bulb in their right hand with their arm stabilized. All subjects had their maximum voluntary contraction (MVC) tested at the start of the experiment and all subsequent forces were scaled accordingly. Subjects were instructed to control an "inflatable" ring as shown as the horizontal bar in Fig. 6 by squeezing the bulb. The ring must move through an undulating tunnel without touching the sides. The required pressure was between 5–15% MVC in order to successfully avoid the sides of the tunnel. Two five-minute trials were performed by both normal subjects (N) and PD subjects off medication (PD). During one trial, subjects were asked to squeeze the bulb (SQ) with right hand alone. In another trial, subjects were asked to squeeze the bulb exactly as before, but in addition press a mouse button intermittently with their left hand when they observed a color change in the ring. We were particularly interested in how subjects fared when they were required to do both movements simultaneously (BO) compared to the SQ condition (Fig. 6), as clinically it is observed that PD patients have difficulty performing simultaneous movements.

### EEG Data Preprocessing

**Figure 7:**
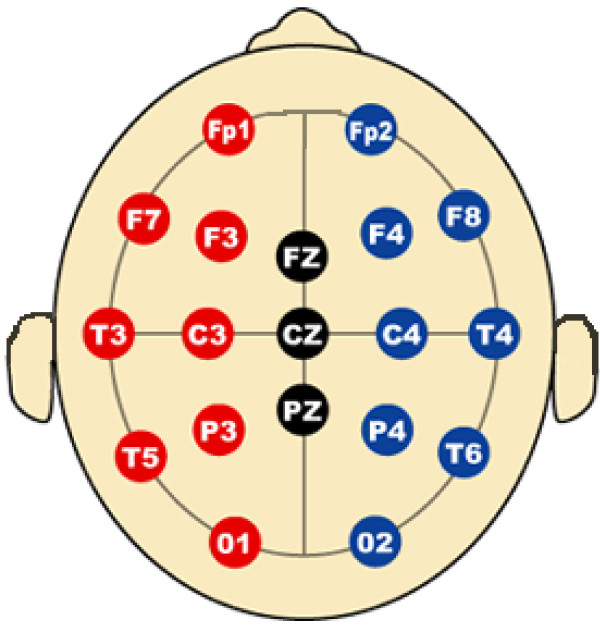
**EEG 10–20 Electrode/Channel Placement**. The channels correspond to the number from 1 to 19 starting from the left to right and top to bottom. Abbreviations: F = frontal, C = central, P = parietal, T = temporal, O = occipital, Fp = frontopolar.

Subjects wore an electrode cap (Electro-Cap International, Eaton, OH) which contained 19 channels and a common mastoid reference (as shown in Fig. 7). The data were collected using a Ceegraph Netlink system from Bio-Logic Systems (Illinois), sampled at 128 Hz and bandpass-filtered between 0.5–55 Hz. After decomposition into temporally independent components with infomax ICA [23], the obviously artifactual components (typically 1 to 2 for each dataset) were screened and removed from the data by visual inspection. Subsequently, two operations are done on the cross-spectrogram of the ICA components: noisy EEG segment removal and task-related EEG segmentation.

#### Noisy EEG Segment Removal

**Figure 8:**
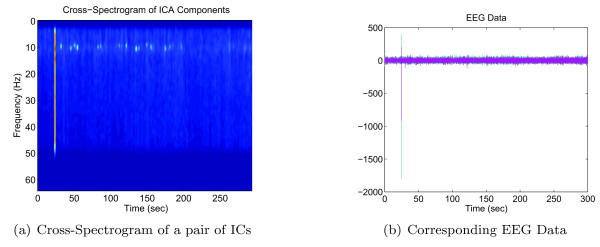
**An example of the cross-spectrogram and EEG**. A broadband artifact (at around 23 s) exists in the cross-spectrogram of the Independent Components (ICs) which reflects the temporal noisy segment in the actual EEG data.

As can be seen in Fig. 8, the broadband artifact right around 23 s of the cross-spectrogram of the ICs reflects a segment in the actual EEG data that is corrupted by noise, and was eliminated by examining the cross spectra of ICs in the 45–55 Hz range.

#### Task-Related EEG Segmentation

The amount that subjects were asked to squeeze was based on two sinusoids with period of 10 and 18 seconds. For the BO condition, the color change occurred every 20 seconds. Therefore, autocorrelations of the cross-spectrogram of the ICs over the three physiologically-relevant frequency bands [21] 5–8 Hz (Theta), 8–12 Hz (Alpha), 12–30 Hz (Beta) that have a peak at 10 seconds or 18–20 seconds are selected for sections of EEG segmentation. Depending on the features of each dataset, approximately five pairs were chosen for each task. Only segments that are above the mean plus the mean absolute deviation are considered as task-related and obtained for further analysis.

**Figure 9:**
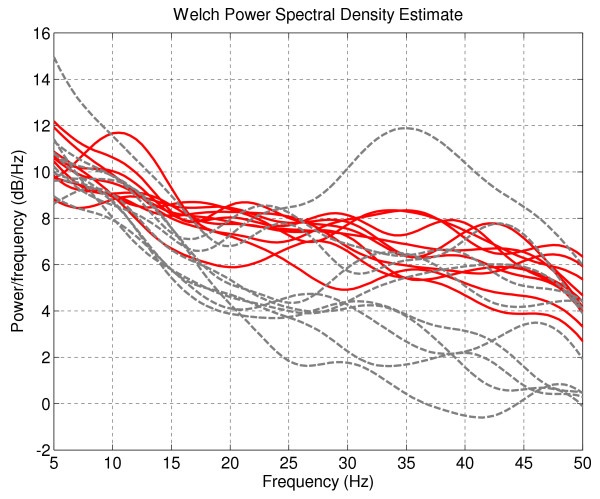
**The power spectral densities (PSDs) of the task-related EEG segments and the non-task-related EEG segments for channel 19-O2**. The solid lines (red) are the PSDs of the task-related EEG segments and the dashed lines (grey) are the PSDs of the non-task-related EEG segments.

To demonstrate the effectiveness of the segmentation method, the power spectral densities (PSDs) of the task-related EEG segments and the non-task-related EEG segments (ie. the ones that are not selected by segmentation for further analysis) were determined. We chose one channel (ie. channel 19-O2) that contained the most number of significant connections (from the network analysis results in Statistical Analysis on MI Network) and investigated its PSD as shown in Fig. 9. The red solid lines are the PSDs of the task-related EEG segments and the grey dashed lines are the PSDs of the non-task-related segments. Fig. 9 clearly illustrates that the variance of the non-task-related segment PSDs is much greater than the one of the task-related segment PSDs. This re-assures us that the proposed segmentation procedure has segmented out similar sections of the EEG, and since the segmentation was based on task-related ICS, the segmented sections correspond to task-related parts of the EEG.

### Mutual Information based Network Analysis

**Figure 10:**
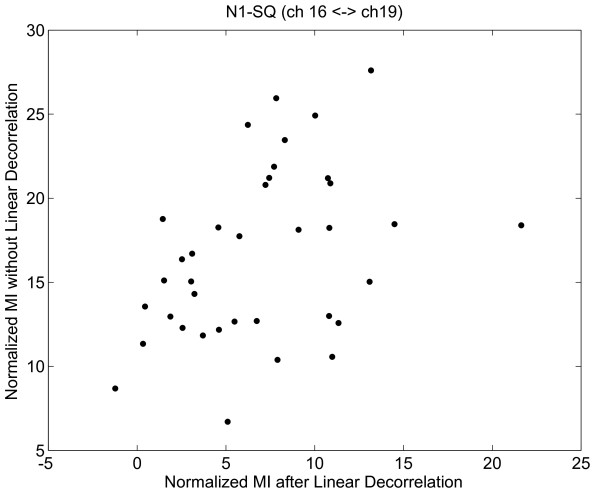
**Scatter plot of MI values before linear decorrelation and after linear decorrelation**. There remains non-linear dependency between channel 16-T6 and channel 19-O2. The MI values are normalized by the corresponding distributions based on permutations.

To assess the importance of non-linear dependencies, which are captured by the proposed MI method, we first linearly decorrelated our data and examined for residual MI values. As one example shown in Fig. 10, we note that there remains dependencies between EEG channels 16-T6 and channel 19-O2 after linear decorrelation, suggesting that MI is a suitable metric to incorporate both linear and nonlinear interactions between EEG channels to derive a more accurate network. Here to make a fair comparison, for each case we derived a null-hypothesis distribution with mean *μ *and standard deviation *σ *from permutations, and then the normalized MI is calculated as  for the scatter plot in Fig. 10.

#### Graphical Theoretical Analysis Applied to the Relevance Network

The MI matrix for each subject is converted to a graph separately, and the means of the cluster coefficient *C *and the shortest path length *L *of the graph within the group (ie. N-SQ, PD-SQ, N-BO, PD-BO) were computed as a function of the threshold *T*.

**Figure 11:**
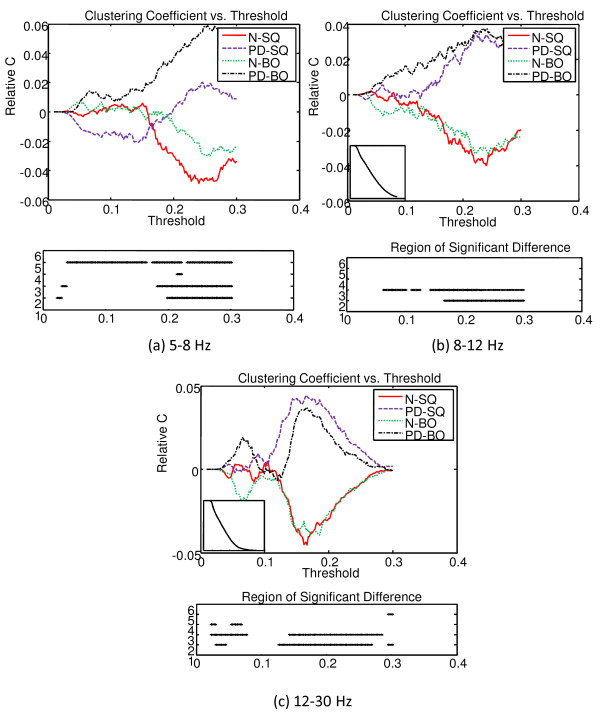
**The deviation of clustering coefficient C of the graph from the overall mean of the four groups as a function threshold T at three frequency bands**. The overall mean of the four groups is presented at the bottom left corner of the top panel. The bottom panel indicates the region that is significantly different between groups (at 2: N vs. PD during SQ; at 3: N vs. PD during BO; at 4: SQ vs. BO for N; at 5: SQ vs. BO for PD).

As *T *is varied from 0.01 to 0.3, the graphs started to break into subgraphs. In addition, at higher *T*, some subjects start to have empty graphs meaning the graphs contain no connection at all. Therefore, when we interpret the results, we need to make note of where those points are and they are summarized in Table 1. Again, we see that the points between N and PD do not differ much because their means are very close. The overall mean *C *of the graph for each group as a function of *T *was computed and compared. Because the means of the four groups (ie. N-SQ, PD-SQ, N-BO, PD-BO) as a function of *T *follow the same pattern and we are more interested in the differences between groups, the deviation from the overall mean of the four groups as a function of *T *is illustrated at the top panel of Fig. 11. The overall mean of the four group as a function of *T *is displayed at the bottom left corner of the top panel. The bottom panel shows the region that is significantly different between groups (at 2: N vs. PD during SQ; at 3: N vs. PD during BO; at 4: SQ vs. BO for N; at 5: SQ vs. BO for PD). The significance of the C between groups is tested with the pair-wise t-test (*p *< 0.05). In general, the intensity of connections within the cluster does not differ significantly between tasks (SQ and BO) within a group. The intensity of connections within the cluster is higher for PD compared to N for SQ task and BO task, especially at higher threshold *T *(*T *> 0.15). The consistently lower *C *seen in N compared to PD across all frequency bands probably reflects the widespread, excessive synchronization seen in PD [34,35]. Unlike prior studies that have emphasized synchronization in the beta band, we have observed excessive synchronization in all bands, especially the theta band.

**Figure 12:**
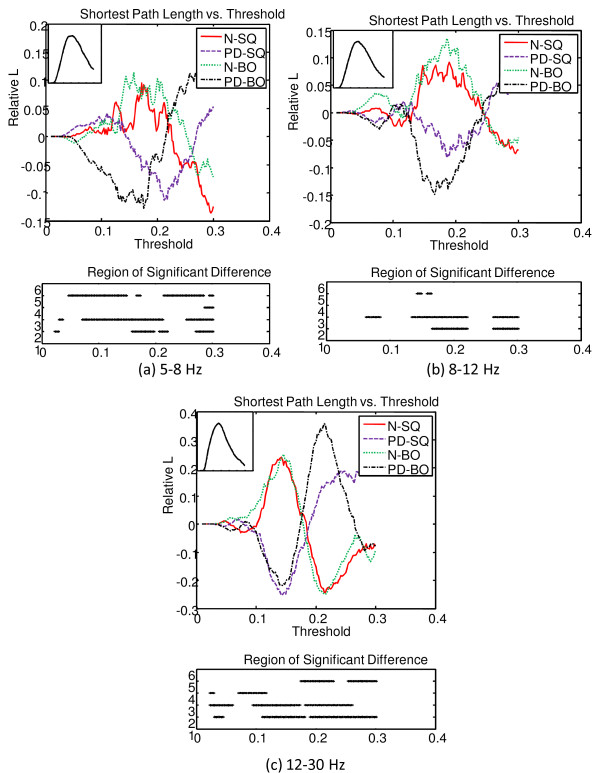
**The deviation of the all shortest path L of the graph from the overall mean of the four groups as a function threshold T at three frequency bands**. The overall mean of the four groups is presented at the top left corner of the top panel. The bottom panel indicates the region that is significantly different between groups (at 2: N vs. PD during SQ; at 3: N vs. PD during BO; at 4: SQ vs. BO for N; at 5: SQ vs. BO for PD).

The top panel of Fig. 12 shows the deviation of *L *from the overall mean of the four groups as a function of the threshold *T*. The overall mean of the four groups is presented at the top left corner of the top panel. The region that is significantly different between groups (denoted at 2: N vs. PD during SQ; at 3: N vs. PD during BO; at 4: SQ vs. BO for N; at 5: SQ vs. BO for PD) is shown at the bottom panel. The *L *of the N group is quite a bit larger than that of the PD group for a threshold between 0.1 to 0.25, but *L *of PD becomes quite a bit larger than that of N after that. However, the magnitude of *L *must be interpreted with caution as the region of significance lies in where subgraphs emerge. Overall, the observation that PD subjects are with larger *C *and shorter *L *compared to N subjects suggests that the graphs for the PD group are more broken up into small, tightly connected clusters.

**Table 1:** Squeeze Task (SQ) and Both Task (BO): Threshold *T *when graphs start to split into subgraphs or become empty graphs

	N-SQ		PD-SQ		N-BO		PD-BO	
	Subgraphs	Empty Graphs	Subgraphs	Empty Graphs	Subgraphs	Empty Graphs	Subgraphs	Empty Graphs

5–8 Hz	0.0600	0.2340	0.0660	0.2380	0.0760	0.2600	0.0600	0.2360
8–12 Hz	0.0520	0.2140	0.0560	0.2120	0.0460	0.1980	0.0580	0.1980
12–30 Hz	0.0500	0.2060	0.0520	0.1660	0.0440	0.1920	0.0540	0.1640

**Figure 13:**
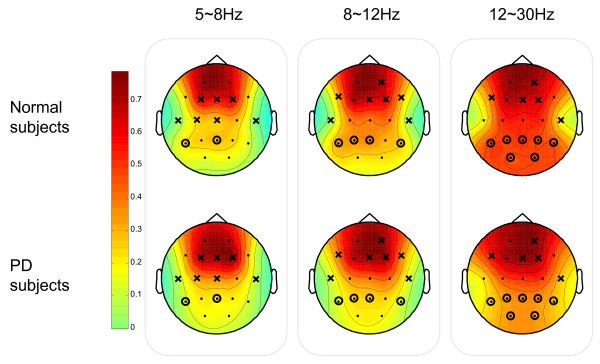
**Squeeze Task (SQ): The graphical representation of the mean clustering coefficient C for each vertex (EEG node) for both Normal (N) and Parkinson groups**. The colorbar displayed vertically indicates the range of the group mean clustering coefficient values for each node. The node that is crossed out indicates that the corresponding group mean *C *of the node for the PD group is significantly greater than the one for the N group. The node that is circled denotes that the corresponding group mean *C *for N is significantly greater than the one for PD.

**Figure 14:**
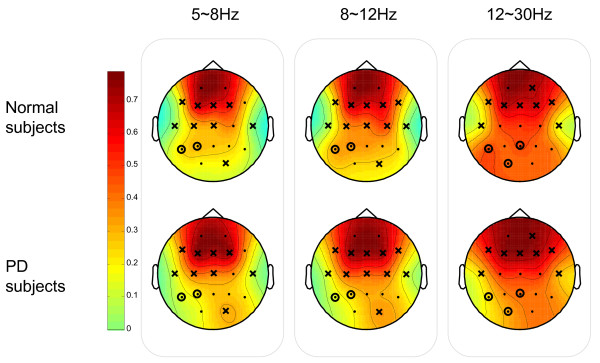
**Both Task (BO): The graphical representation of the mean clustering coefficient *C *for each vertex for both Normal (N) and Parkinson groups**. The node that is crossed out indicates that the corresponding group mean *C *of the node for the PD group is significantly greater than the one for the N group. The node that is circled denotes that the corresponding group mean *C *per vertex for N is significantly greater than the one for PD.

In order to gain insight into the location distribution of the clusters, the graphical representations of the overall group mean clustering coefficient *C *for each vertex (i.e. EEG node) are illustrated in Fig. 13 and Fig. 14 for SQ and BO task, respectively. As mentioned earlier, we study three physiologically-relevant frequency bands: 5–8 Hz (Theta), 8–12 Hz (Alpha), 12–30 Hz (Beta). For both the N and PD groups, the images of the group mean clustering coefficient values of 19-nodes are displayed using the jet colormap. In the figures, the node that is crossed out indicates that the corresponding mean *C *for the PD group is significantly greater than that of the N group. The node that is circled denotes that the corresponding mean *C *of that vertex for the N group is significantly greater than the one for the PD group. The significance between the mean *C *per vertex for the N and the PD groups is tested by pair-wise T-test (*p *< 0.05) and the details are shown in Table 2 and 3. Only the channels that have any significant difference between groups are shown in the tables. It can be seen that the PD group has more heavily connected clusters at the frontal and motor cortex of the brain over all frequencies. On the other hand, the N group has more heavily connected clusters at the posterior region of the brain over all frequencies.

**Table 2:** Squeeze Task (SQ): Clustering coefficient *C *per vertex and the p-value of the pair-wise T-test between Normal and Parkinson's subjects.

	5–8 Hz			8–12 Hz			12–30 Hz		
Channel #	N mean	PD mean	P-value	N mean	PD mean	P-value	N mean	PD mean	P-value

2-Fp2	0.5786	0.6075	4.42E-01	0.6229	0.7133	4.97E-03	0.6982	0.7660	6.75E-03
3-F7	0.2106	0.2770	9.33E-02	0.2705	0.3638	2.58E-02	0.3961	0.5643	5.95E-05
4-F3	0.5290	0.6062	3.28E-02	0.6170	0.6470	3.35E-01	0.6781	0.7039	2.37E-01
5-FZ	0.4791	0.6041	6.92E-06	0.5648	0.6767	6.15E-06	0.6439	0.7440	2.82E-07
6-F4	0.5204	0.6574	8.34E-05	0.5363	0.6728	4.11E-06	0.6148	0.7013	1.39E-04
7-F8	0.1958	0.2478	1.40E-01	0.2806	0.3679	2.95E-02	0.4731	0.4819	8.31E-01
8-T3	0.0159	0.0630	7.14E-03	0.0312	0.1073	1.20E-03	0.1767	0.2333	1.10E-01
9-C3	0.2138	0.2895	2.82E-02	0.3395	0.3392	9.92E-01	0.5177	0.4640	7.19E-02
10-CZ	0.2229	0.3275	9.49E-04	0.3466	0.3913	1.35E-01	0.5209	0.5234	9.20E-01
12-T4	0.0054	0.0739	2.38E-04	0.0453	0.1346	8.02E-04	0.1734	0.2746	4.57E-03
13-T5	0.1844	0.1034	1.06E-02	0.2824	0.1384	7.93E-05	0.4799	0.2557	8.59E-08
14-P3	0.2241	0.1917	3.05E-01	0.3117	0.2322	1.17E-02	0.4765	0.3414	7.11E-06
15-PZ	0.2409	0.1787	3.92E-02	0.3340	0.2310	8.52E-04	0.5111	0.3665	8.04E-07
16-P4	0.2139	0.1838	3.25E-01	0.2691	0.2423	3.85E-01	0.4560	0.3563	6.30E-04
17-T6	0.1123	0.1057	8.14E-01	0.2228	0.1265	3.59E-03	0.4521	0.2778	2.94E-05
18-O1	0.1310	0.1537	4.61E-01	0.2029	0.1955	8.25E-01	0.4547	0.3436	4.26E-03
19-O2	0.1428	0.1402	9.35E-01	0.2083	0.1900	5.93E-01	0.4615	0.3422	1.86E-03

(*T *= 0.2 for 5–8 Hz; *T *= 0.18 for 8–12 Hz; *T *= 0.15 for 12–30 Hz)

**Table 3:** Both Task (BO): Clustering coefficient *C *per vertex and the p-value of the pair-wise T-test between Normal and Parkinson's subjects. (*T *= 0.2 for 5–8 Hz; *T *= 0.18 for 8–12 Hz; *T *= 0.15 for 12–30 Hz)

	5–8 Hz			8–12 Hz			12–30 Hz		
Channel #	N mean	PD mean	P-value	N mean	PD mean	P-value	N mean	PD mean	P-value

2-Fp2	0.5988	0.6179	5.83E-01	0.6801	0.7089	3.26E-01	0.7158	0.7630	2.37E-02
3-F7	0.1758	0.3215	8.66E-05	0.2286	0.3980	1.72E-05	0.3552	0.5811	1.91E-08
4-F3	0.5665	0.6533	8.77E-03	0.6233	0.6993	9.53E-03	0.6844	0.7434	5.65E-03
5-FZ	0.5188	0.6053	1.40E-03	0.5661	0.6955	1.14E-08	0.6334	0.7611	4.61E-12
6-F4	0.4917	0.6331	1.03E-05	0.5972	0.6808	1.75E-03	0.6668	0.7204	7.50E-03
7-F8	0.2554	0.3190	9.12E-02	0.3399	0.4300	2.19E-02	0.5210	0.6103	1.71E-02
8-T3	0.0237	0.1047	2.86E-04	0.0520	0.1321	2.12E-03	0.1746	0.2498	4.09E-02
9-C3	0.2392	0.3473	1.54E-03	0.3431	0.4263	1.87E-02	0.4820	0.5116	3.13E-01
10-CZ	0.2741	0.3395	2.41E-02	0.3627	0.4190	4.90E-02	0.5087	0.5213	6.16E-01
11-C4	0.3185	0.3149	9.15E-01	0.4567	0.4056	1.25E-01	0.4975	0.5294	2.26E-01
12-T4	0.0133	0.0900	4.02E-05	0.0580	0.1366	1.70E-03	0.1689	0.2995	2.04E-04
13-T5	0.2043	0.1184	6.77E-03	0.3055	0.1369	2.05E-06	0.4810	0.2283	3.19E-10
14-P3	0.2537	0.1812	1.67E-02	0.3270	0.2357	2.92E-03	0.4320	0.3747	5.79E-02
15-PZ	0.2377	0.2390	9.63E-01	0.3323	0.3010	3.00E-01	0.4923	0.4195	8.46E-03
18-O1	0.1629	0.1489	6.29E-01	0.2441	0.1898	9.16E-02	0.4316	0.3235	3.39E-03
19-O2	0.1652	0.2749	2.02E-03	0.2322	0.3291	8.20E-03	0.4322	0.4095	5.38E-01

#### Statistical Analysis on MI Network

**Figure 15:**
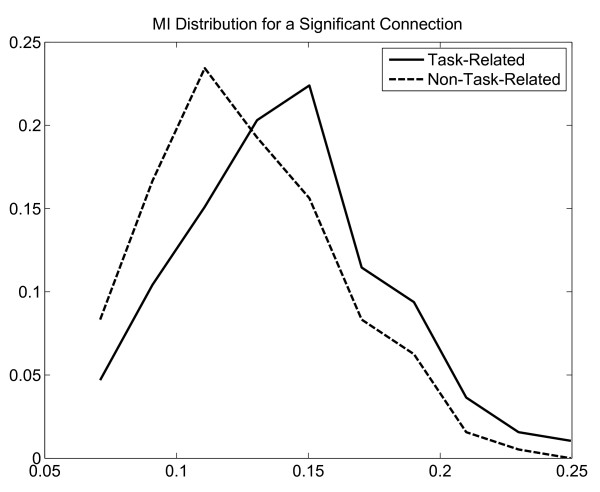
**An example of MI distribution of a significant connection: Task-related vs. Non-task-related**.

To further demonstrate the importance of segmentation, we calculated MI networks for both segmented and unsegmented data. The majority of the connections indicates that the MI values based on the task-related segments are significantly greater than the ones based on the non-task-related segments for all networks. One example of the MI distribution of a significant connection is illustrated in Fig. 15. As implied from the networks, there is a shift in the mean of the MI values between the task-related segments and the non-task-related segments.

**Figure 16:**
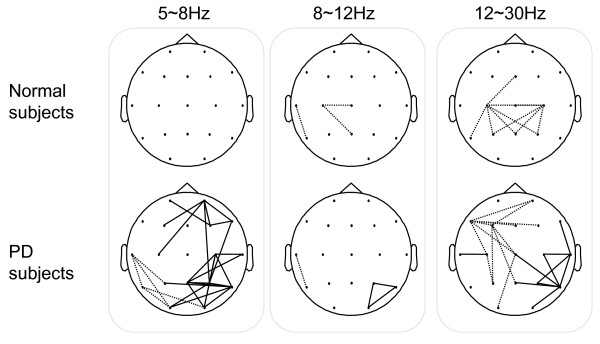
**The MI Networks for Intra-Group Analysis: Squeeze task (SQ) vs. Both task (BO)**. The solid lines denote that the MI values of the BO task are significantly greater than the ones for the SQ task. The dotted lines represent the converse.

To investigate the effect of the disease, the MI networks were computed for the intra-group analysis (SQ vs. BO) and the inter-group analysis (N vs. PD). The graphical results at three different frequency bands for Normals and PD subjects are presented in Fig. 16. The solid lines denote that the MI values of the BO task are significantly greater than the ones for the SQ task and the dotted lines represent the converse condition. The results suggest that PD subjects are unable to independently recruit different areas of the brain while performing simultaneous tasks, but instead simultaneously recruit focal islands of increased synchrony.

**Figure 17:**
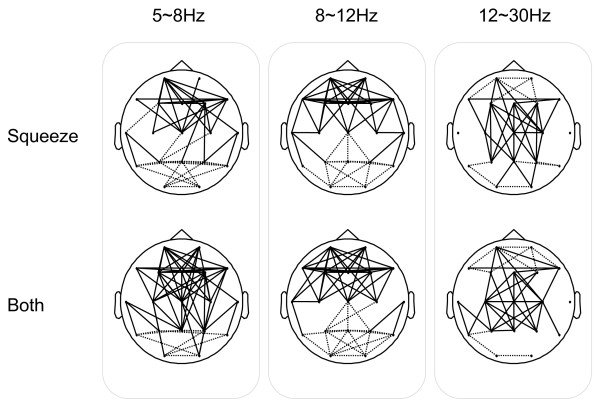
**The MI Networks for Inter-Group Analysis: Normal Subjects (N) vs. Parkinson's subjects**. The solid lines represent that the MI values of PD are significantly larger than the ones of N. On the other hand, the dotted lines show that the MI values of N are significantly greater than the ones of PD.

We also investigated the results for inter-group analysis at three different frequencies (Fig. 17). We observe higher MI values in the frontal region at lower and medium frequency bands and motor cortex at higher frequency band in PD which coincide with the finding in the previous graphical theoretical analysis.

## Conclusion

This paper proposed a novel segmentation, mutual information network framework for EEG connectivity analysis for subjects performing a motor task. The greatest EEG changes during motor performance are typically event-related synchronization/desynchronization: EEG responses are not phase-locked to motor performance, but rather tend to be associated with augmentation or attenuation of specific frequency bands. This means that standard methods of averaging the EEG time-locked to the motor performance will tend to be inaccurate, necessitating the use of alternate methods. In addition, ERS/ERD is typically investigated in univariate fashion, where each EEG channel is examined independently for altered localized neuronal synchrony resulting in changes in the frequency spectra at that channel. Here we have used the cross-spectrum of ICs as a marker for segmentation. This has multiple benefits: first, consistent with ERS/ERD, it examines the data in the frequency domain, second, it allows the examination of multiple channels simultaneously, as each IC will consist of a linear weighting of all channels, and lastly, it will allow unmixing of the raw data so that task related activity can be extracted from ongoing background brain rhythms. After segmentation, we used mutual information to measure both linear and nonlinear dependencies, without assuming strict phase locking of signals. This allowed us to create a relevance network suitable for graph-theoretic analysis methods, and an MI network which further incorporated the magnitude of the MI at each channel pair to allow statistical analysis with ANOVA.

The proposed method provided several novel insights into abnormalities in PD subjects. The well known clinical observation of difficulty in performing simultaneous movements [12] appears to be related to an inability to recruit different brain areas independently. When normal subjects were asked to perform tasks with two hands compared to a task with one hand, there was no significant difference in the theta bands, and only mild changes in connectivity in the alpha and beta bands (Fig. 16). In contrast, when PD subjects attempted to perform simultaneous movements, they appeared unable to recruit different brain areas independently, resulting multiple areas of synchronization in the theta range, and also in the beta range. These results appear novel, as previous research has emphasized excessive synchronization in the beta range in PD [36].

Additionally, the widespread synchrony that is normally already seen during regular unimanual or bimanual performance was of a different form in PD (Figs. 11, 12, and 17). PD subjects tended to have higher cluster coefficients, *C*, and lower shortest path lengths, *L *suggesting focal clusters of synchronous activity. Taken together, these results suggest that normal subjects can synchronously activate broad areas or cortex independently in response to differing task demands. In contrast, PD subjects appear to have islands of hypersynchronicity that cannot be recruited independently. This is consistent with known biology of PD, where bradykinesia is most likely the result of "noisy" basal ganglia input to the frontal cortex and appears to critically depend upon dopamine depletion [37] as would be seen in the PD subjects off medication in this study. The higher MI values in the frontal region at lower and medium frequency bands and motor cortex at higher frequency in PD (Fig. 17) are also consistent with the cortical regions that receive output from the basal ganglia. Since the EEG is normally assumed to reflect cortical activity, isolation of clear abnormalities from PD subjects who have predominantly basal ganglia dysfunction and preserved cortical function is notable.

Although we suggest that our results demonstrate strong evidence to support the proposed framework as a tool to study EEG signals, there are nevertheless limitations of the proposed method. For example, currently only pair-wise MI, one particular case of calculating the MI between *M *random variables, is investigated. In a more general presentation, the corresponding *M*–dimensional MI can be defined. Since pairwise independence does not necessarily imply global independence, *M*–dimensional MI may reveal additional information from that of pair-wise MI, and thus may be a fruitful avenue to explore in the future for EEG analysis. Similarly, pair-wise MI may suffer from a high false discovery rate, i.e. nodes are erroneously associated while in truth they only indirectly interact through one or more other nodes. Therefore, to prune the reconstructed network of such false positives, we can extend the current work by exploring the concept of conditional mutual information (CMI) instead. Also, in the current approach, we did not consider the temporal information embedded in the time-series EEG data. As one future work, we intend to introduce temporality into the proposed MI network construction.

## Competing interests

The authors declare that they have no competing interests.

## Authors' contributions

ZW proposed the main ideas, checked the method procedure, and participated in the data analysis and manuscript writing. PL participated in the data acquisition and algorithm implementation, analyzed the data, and drafted the manuscript. MM organized the study, provided the EEG data, and helped to interpret the results and draft the manuscript. All authors read and approved the final manuscript.
